# Reviews, expert opinions, consensus statements, position papers, protocols, and evidence-based guidelines: what are their roles in clinical practice?

**DOI:** 10.1590/0102-67202025000057e1926

**Published:** 2026-03-09

**Authors:** Francisco TUSTUMI, Lucia CALTHORPE, Felipe José Fernández COIMBRA, Adnan ALSEIDI

**Affiliations:** 1 Universidade de São Paulo, Faculty of Medicine, Department of Gastroenterology – São Paulo (SP), Brazil.; 2 University of California, Division of Surgical Oncology, Section of Hepatopancreaticobiliary Surgery – San Francisco (CA), USA.; 3 A.C. Camargo Cancer Center, Department of Surgical Oncology – São Paulo (SP), Brazil.

**Keywords:** Consensus, Guideline, Guidelines as Topic, Publication, Evidence-Based Practice, Consenso, Guia, Guias como Assunto, Publicações, Prática Clínica Baseada em Evidências

## Abstract

The growth of primary research in digestive surgery has led to an increasing need for structured interpretation and application to clinical practice. Guidance documents have become essential tools for decision-making. However, the terminology surrounding clinical guidance documents is frequently inconsistent, and different formats are often used interchangeably. Although review articles synthesize available evidence, they do not constitute clinical guidance unless their findings are interpreted through a framework explicitly oriented toward patient care, applicability, and decision-making. This review article clarifies the conceptual distinctions between expert opinion, consensus statements, position papers, protocols, and evidence-based guidelines. No single guidance format is universally superior, and each has its appropriate context. In digestive surgery, where randomized trials are often difficult to perform and evidence gaps remain frequent, structured interpretation is indispensable. As technology advances and real-world data become increasingly available, new methodological tools — including artificial intelligence and living systematic reviews — may enhance reproducibility and accelerate updates. Ultimately, clearer distinctions among guide orientations and greater methodological transparency may improve clinical judgment, stimulate research, and strengthen patient care in gastrointestinal surgery.

## ARTICLE HIGHLIGHTS

Clinical practice guidance documents differ in purpose, methodology, and level of evidence. They should not be used interchangeably.

Evidence-based guidelines rely on systematic review but require time, expertise, and substantial resources.

Structured consensus is especially valuable when evidence is limited or conflicting.

Protocols and position papers can evolve toward stronger evidence-based frameworks when prospectively validated, as seen with ERAS (Enhanced Recovery After Surgery) and ACERTO (*ACEleração da Recuperação TOtal Pós-operatória*).

## Summary

**CENTRAL MESSAGE:**

Clinical practice guidance documents are not all the same. In digestive surgery, evidence-based guidelines, consensus statements, position papers, and protocols each serve different purposes, and the distinctions between them should be clear. Recognizing the strengths and limitations of each format is essential to improving decision-making, guiding research, and ultimately benefiting our patients.

**PERSPECTIVES:**

Digestive surgery evolves rapidly, and traditional publication models may not keep pace with emerging technologies and clinical demands. Future guidance documents are expected to incorporate artificial intelligence for evidence screening, living systematic reviews for continuous updates, and transparent digital repositories for open access to consensus voting and dissent. National registries and real-world data will complement trials where randomized evidence is scarce.

## VISUAL ABSTRACT

**Figure:**
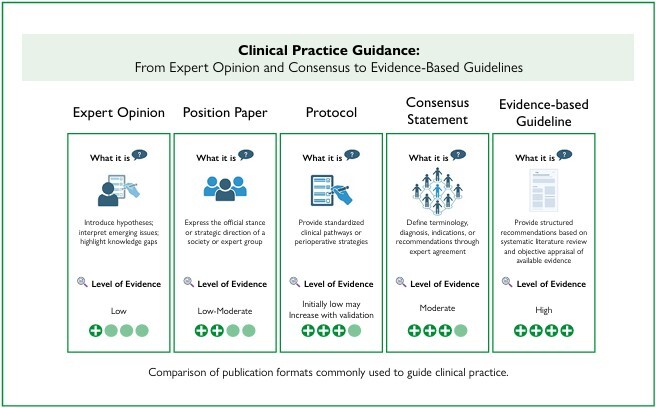

## RESUMO

O crescimento da pesquisa primária em cirurgia digestiva levou a uma necessidade crescente de interpretação estruturada e aplicação à prática clínica. Os artigos de orientação tornaram-se ferramentas essenciais para a tomada de decisões. No entanto, a terminologia em torno desses artugis é frequentemente inconsistente, e diferentes formatos são muitas vezes usados de forma intercambiável. Embora os artigos de revisão sintetizem as evidências disponíveis, eles não constituem orientação clínica, a menos que suas descobertas sejam interpretadas por meio de uma estrutura explicitamente orientada para o cuidado do paciente, aplicabilidade e tomada de decisões. Este artigo de revisão esclarece as distinções conceituais entre opinião de especialistas, declarações de consenso, documentos de posição, protocolos e diretrizes baseadas em evidências. Nenhum formato de orientação é universalmente superior e cada um tem seu contexto apropriado. Em cirurgia digestiva, onde os ensaios randomizados são frequentemente difíceis de realizar e as lacunas de evidências permanecem frequentes, a interpretação estruturada é indispensável. À medida que a tecnologia avança e os dados do mundo real se tornam cada vez mais disponíveis, novas ferramentas metodológicas — incluindo inteligência artificial e revisões sistemáticas contínuas — podem aprimorar a reprodutibilidade e acelerar as atualizações. Em última análise, distinções mais claras entre os guias de orientação e maior transparência metodológica podem melhorar o julgamento clínico, estimular a pesquisa e fortalecer o cuidado ao paciente em cirurgia gastrointestinal.

## INTRODUCTION

 The expansion of primary research in digestive surgery has been remarkable over the past two decades. Randomized clinical trials, observational cohorts, and registry-based studies have become increasingly common^13,21^. Nonetheless, these studies often differ significantly in methodology, include heterogeneous patient populations, or are conducted in highly specialized centers that may not reflect worldwide surgical practice. As a consequence, results frequently conflict with one another, creating uncertainty in daily decision-making and raising doubts among clinicians who must decide how to apply these findings in real-world settings^8^. These challenges are especially evident in gastrointestinal surgery, where therapeutic decisions depend not only on disease characteristics but also on surgical expertise, institutional resources, and the timing of presentation. 

 It is in this context that clinical practice guidance documents have gained importance over the past decades. Rather than simply summarizing available literature, these documents aim to orient clinical practice by addressing specific and relevant questions. Usually, they seek to clarify what should be done, which patients benefit most, and under what degree of certainty recommendations can be applied. Today, a wide spectrum of group-based publications, including consensus statements, position papers, evidence-based guidelines, protocols, and expert opinions, play a central role in guiding surgical management worldwide. These guidance documents are not legally binding, and physicians may depart from them when judged clinically appropriate. However, such deviations should be justified and documented^10^. Unlike directives, guidance documents are intended to inform, rather than dictate decision-making, particularly in complex surgical scenarios where standardized recommendations must be weighed against patient-specific factors. 

 However, the terminology applied to these publications is often inconsistent, and even large scientific societies have used the term "guideline" to describe documents that do not meet formal methodological standards. To address this concern, international frameworks — such as the classification proposed by the Association of the Scientific Medical Societies in Germany (AWMF) — have introduced structured categories ranging from purely experience-based recommendations (S1) to fully evidence-based and consensus-driven guidelines (S3). S1 documents rely solely on expert opinion; S2K are consensus-based but lack systematic evidence synthesis; S2e involve structured evidence collection without a consensus process; and only S3 guidelines combine systematic review methodology with a predefined consensus protocol. According to this model, only S3 guidelines should be regarded as fully evidence-based and methodologically comprehensive^18^. 

 Clinical practice guidance documents differ widely in purpose, methodology, and the level of evidence they present (Table 1). Recognizing these differences is essential for surgeons, reviewers, and editors — especially in digestive surgery, where evidence gaps, technological heterogeneity, and clinical variability are common. This manuscript clarifies the conceptual distinctions among existing types of guidance documents, discusses their roles within digestive surgery, and emphasizes the importance of methodological transparency when interpreting or developing these publications. Ultimately, a clearer understanding of their nature, strengths, and limitations may improve both clinical decision-making and the quality of future surgical research. 

**Table 1:** Comparison of publication formats commonly used to guide clinical practice.

Type of article	Main purpose and characteristics	Typical methodology	Level of evidence	Advantages and roles	Disadvantages
Expert opinion	Introduce hypotheses; interpret emerging issues; highlight knowledge gaps	Based on individual or group experience; no structured method	Low	Useful when evidence is scarce; may propose novel perspectives; stimulates discussion	Not reproducible; highly subjective; vulnerable to bias; low credibility
Position paper	Express the official stance or strategic direction of a society or expert group	Argumentative review; include selective evidence and expert interpretation	Low-Moderate	Clarifies conceptual distinctions and institutional strategies; may influence policy or practice	Potential for selective citation; limited generalizability
Protocol	Provide standardized clinical pathways or perioperative strategies	Structured procedural framework	Initially low; may increase with validation	Facilitates implementation; enables multidisciplinary collaboration; may generate new evidence when evaluated prospectively	Often based on expert consensus alone; requires validation; may not be generalizable
Consensus statement	Define terminology, diagnosis, indications, or recommendations through expert agreement	Delphi, nominal group technique, RAND-UCLA appropriateness method, consensus conference	Moderate	Supported by available data and collective expert interpretation; transparent methodology; applicable when evidence is insufficient; practical for real-world decision-making	Risk of subjectivity; variability among experts; results change if panel changes; less methodological rigor than evidence-based guidelines
Evidence-based guideline	Provide structured recommendations based on a systematic structure	Systematic review + validated appraisal frameworks (e.g., GRADE, Oxford, USPSTF, NICE, SIGN)	High	Strong evidence base; hierarchical structure; regularly updated; promotes standardization; reproducible and auditable	Requires substantial time and resources; may be complex to interpret; may not fully reflect individual variability or regional constraints

RAND-UCLA: Research and Analysis – University of California Los Angeles; USPSTF: U.S. Preventive Services Task Force; NICE: National Institute for Health and Care Excellence; SIGN: Scottish Intercollegiate Guidelines Network; GRADE: Grading of Recommendations Assessment, Development and Evaluation.

### Reviews and guidance documents

 Generally speaking, clinical guidance documents are fundamentally grounded in some form of literature synthesis. Review papers, whether systematic or narrative, serve as the analytical substrate upon which interpretations and recommendations are later constructed. However, the presence of a review does not, by itself, create a guidance document. Many reviews explore basic science, epidemiology, or surgical techniques without aiming to orient clinical decisions, while critical reviews may highlight controversies or interpret data without offering prescriptive recommendations. In this sense, review papers provide the evidentiary foundation, whereas guidance documents add a further layer of structured judgment, explicit methodology, and clinical intent. For a review to evolve into a true guidance document, two additional elements are indispensable: first, the evidence must be interpreted through a structured decision-making framework — one that considers the strength of recommendations, the balance between benefits and harms, implications, and issues of feasibility; second, the intent of the document must be explicitly directed toward informing clinical practice rather than merely summarizing evidence. This distinction is crucial: guidance requires evidence, but evidence synthesis alone does not constitute clinical guidance. 

### Opinion versus evidence

 Broadly speaking, guidance documents can be conceptually grouped into those primarily based on evidence and those structured around expert opinion. Evidence-based documents are fundamentally grounded in systematic literature reviews, which may include quantitative synthesis, such as meta-analysis, or qualitative synthesis when data are heterogeneous. Systematic reviews follow a formal and transparent methodology that typically begins with the prospective registration of a protocol (e.g., PROSPERO; https://www.crd.york.ac.uk/prospero/), formulation of a focused research question, and development of a comprehensive search strategy across multiple databases with predefined inclusion and exclusion criteria. Study selection is usually performed in duplicate, followed by standardized data extraction using piloted forms to minimize reviewer bias. The next steps involve a structured risk-of-bias assessment using validated tools, depending on the study design. When feasible, meta-analysis is conducted with appropriate models and heterogeneity evaluation; when not feasible, synthesis remains qualitative and narrative^14^. Cochrane tools remain central to this process, and frameworks derived from them, such as GRADE (Grading of Recommendations Assessment, Development, and Evaluation), allow researchers to move beyond a simple hierarchy of evidence and evaluate imprecision, inconsistency, indirectness, and publication bias^7^. In theory, any researcher with appropriate training in systematic review methodology can undertake such work, which should remain independent of clinical expertise. Methodological rigor is crucial to ensure reproducibility: different individuals following the same protocol should reach similar conclusions. However, the transition from evidence synthesis to guideline recommendations requires explicit judgment: guideline panels must rate the strength of recommendations, consider the balance between benefits and harms, assess patient values and preferences, evaluate resource use, and examine feasibility and equity implications. 

 Within this context, evidence-based clinical practice guidelines represent the highest level of structured, group-based recommendations. Their development is driven by clinically relevant questions and systematic approaches to the literature. When randomized trials are unavailable, observational studies are used, always with explicit acknowledgment of the limitations inherent to lower-certainty evidence. These principles explain why evidence-based guidelines are valued: they provide transparency, allow scrutiny, and make the reasoning process visible to the reader. However, they demand time, expertise, and dedicated resources. As evidence evolves, recommendations may already be outdated by the time guidelines are published, requiring continuous revision^22^. 

 In an effort to improve clarity and usability, many guidelines incorporate visual algorithms or flowcharts to illustrate diagnostic workups or therapeutic pathways. These schematic tools aim to facilitate reader comprehension by translating complex recommendations into compartmentalized decision trees. However, most flowcharts rely on dichotomous decisions, whereas real-world care is influenced by multidimensional variables that rarely fit into binary choices. This limitation becomes especially evident in digestive surgery, where shared decision-making must incorporate individual preferences, comorbidities, frailty, social context, and institutional capacity. 

 The process of moving from evidence to recommendation always requires judgment and interpretation. Even when evidence is high-quality, it does not automatically dictate clinical decisions. The growing emphasis on precision medicine, especially in oncology, where molecular biomarkers increasingly guide treatment, illustrates this contrast. Precision care moves beyond generalized flowcharts by recognizing that individual biological and clinical characteristics can substantially alter management decisions. 

 Digestive surgery presents additional limitations for purely evidence-based approaches. Randomized controlled trials are often difficult to perform and nearly impossible to blind. Some attempts have been made, such as in the LEOPARD trial (Looking at EVAR Outcomes by Primary Analysis of Randomized Data), in which wound dressings were used to conceal the operative technique from patients^5^. Yet such strategies raise ethical concerns. Moreover, unlike pharmacological treatments, surgical procedures depend on the skill, training, and experience of the operator. Variability among surgeons and institutions is intrinsic to surgical care. Consequently, even randomized trials require careful interpretation. Furthermore, many surgical diseases are inherently less frequent than chronic clinical conditions, especially because surgical treatment is typically employed when clinical therapy has failed or is not feasible. In many cases of gastrointestinal cancer, patients are diagnosed at an advanced stage when curative surgical resection is no longer feasible^2^. These epidemiological realities reduce the feasibility of randomized trials and often result in small sample sizes with low statistical power. Additionally, digestive surgery rapidly incorporates new technologies that evolve faster than clinical trials can be completed. In early robotic trials, for instance, some studies were published using the da Vinci Si platform after it had already been discontinued^24^. These challenges explain why evidence alone is often insufficient and why expert interpretation of available data becomes essential. 

 Interpretation plays a central role even when randomized trials exist. All evidence, whether derived from trials or observational studies, requires interpretation. Relying on expert opinion may initially seem incompatible with evidence-based medicine, and its limitations are well recognized. Yet the intention is not to replace evidence with authority, but to incorporate clinical expertise thoughtfully when interpreting the available data^20^. Guideline panels must consider whether risk of bias compromises confidence in results, whether confidence intervals are vast, whether findings can be generalized to surgical populations with greater complexity, and whether unexplained heterogeneity precludes strong inference. In some scenarios, conclusions are obvious, but in most cases, interpretation demands collective and reasoned judgment. This is where expert opinion methodologies become relevant. 

 Expert opinion emerges particularly when evidence is scarce, contradictory, or insufficient to address urgent clinical questions. Although it is not based on a formal methodological framework, expert opinion is rarely generated in isolation from the literature. Critical reviews, non-systematic yet analytically rigorous evaluations of available studies, often serve as the intellectual foundation upon which expert perspectives are formed. These reviews synthesize strengths, limitations, gaps, and inconsistencies in the evidence, allowing experts to contextualize findings and extrapolate beyond what the data directly demonstrate. In this sense, critical reviews do not constitute guidance themselves, as they may address themes that do not translate directly into clinical decision-making, but they provide the interpretative substrate for authoritative judgment. However, because expert opinion ultimately depends on individual interpretation, it lacks reproducibility and remains vulnerable to bias. Consensus, therefore, becomes the next step. It is an effort to organize expert interpretation through a shared framework. Instead of isolated opinions, it seeks agreement among multiple specialists and formalizes collective judgment. 

 Consensus statements differ fundamentally from expert opinion because they rely on predefined methodological steps and aim to be reproducible. They seek to transform individual viewpoints into a coherent collective interpretation of available evidence. In many consensus initiatives, structured evidence syntheses, such as scoping reviews, rapid reviews, or targeted literature searches, are performed beforehand to map existing knowledge, delineate areas of uncertainty, and ensure that expert deliberation is anchored in a transparent and comprehensive evidentiary foundation. Scoping reviews follow a structured process to map available evidence, typically including protocol registration and comprehensive searches to identify concepts, definitions, and knowledge gaps. Rapid reviews use abbreviated systematic review methods, such as restricted search strategies, single-reviewer screening with verification, and focused extraction, to generate evidence summaries within short time frames. Although these reviews do not replace systematic reviews, they frequently serve as a pragmatic substrate for consensus development, particularly when time constraints, evolving technologies, or heterogeneous data preclude full systematic synthesis. Their flexibility makes them adaptable to real clinical situations, although this also introduces the possibility of subjectivity and selective citation. 

 Consensuses should not be mistaken for unstructured opinion, as validated methods exist to guide their development. Among these, the Delphi method is the most extensively used. It relies on anonymous voting across iterative rounds, predefined agreement thresholds, and controlled feedback to refine responses until opinion stability is reached. Its methodological structure typically includes careful selection of experts, development of structured questionnaires, iterative scoring of statements, and explicit criteria for defining consensus, ensuring both transparency and reproducibility. The nominal group technique depends on moderated face-to-face discussion and is often combined with Delphi iterations. The RAND-UCLA (Research and Analysis – University of California Los Angeles) appropriateness method links appraisal of evidence with expert judgment using two complementary panels and has been applied to evaluate the appropriateness of gastrointestinal procedures. Consensus development conferences bring together multidisciplinary experts to deliberate on specific topics under predefined discussion rules^11,12,16^. 

 Consensus statements in digestive surgery include influential documents that have shaped current practice. The Tokyo guidelines for acute cholangitis and cholecystitis introduced severity grading and treatment algorithms that have been widely adopted^26^. The European Hernia Society has provided relevant practical approaches for hernia management^23^. The São Paulo International Consensus on Minimally Invasive Pancreatic Surgery for Cancer relied on Delphi methodology to establish agreement thresholds among experienced HPB (Hepato Bilio Pancreatic) surgeons across multiple continents^25^. In recent years, several other consensus initiatives in digestive surgery have gained relevance, contributing to standardized definitions of anastomotic leaks, management pathways for colorectal and upper gastrointestinal cancers, liver resection strategies, and bariatric procedures, among others^9,15,17,19^. 

 When robust evidence is available, consensus recommendations may approximate those of evidence-based guidelines. When the evidence is weak or inconsistent, evidence-based guidelines often rely on observational studies with low certainty, whereas consensus statements rely on structured expert judgment. In practice, surgeons frequently encounter situations in which delay is not possible and randomized evidence is lacking. In such settings, expert consensus provides clinical guidance that bridges the gap between evidence and action. However, consensus carries inherent limitations. It may lack methodological rigor, may be influenced by expert selection, and may not be reproducible if panel composition changes. 

 Beyond consensus and evidence-based guidelines, other clinical guidance document formats play distinct roles in surgical practice. Position papers represent the formal stance of a scientific society or expert group. They are not designed to synthesize the available literature exhaustively but to guide practice based on collective clinical judgment and strategic priorities, often reflecting national realities or pragmatic constraints. In digestive surgery, the Brazilian Archives of Digestive Surgery has produced influential position papers addressing perioperative care, antibiotic prophylaxis, and the management of acute cholecystitis^4,6^. 

 Protocols represent another format. Many begin as expert-driven initiatives — often driven by the need to standardize perioperative care within a hospital or network. When systematically implemented, prospectively evaluated, and refined through multicenter collaboration, protocols can transition toward an evidence-based framework. The ERAS (Enhanced Recovery After Surgery) protocols exemplify this evolution, moving from expert consensus to rigorously validated strategies with measurable outcomes. In Brazil, the ACERTO (*ACEleração da Recuperação TOtal Pós-operatória*) project followed a similar trajectory: it began as a structured approach to perioperative optimization and, through implementation and academic dissemination, contributed to a stronger culture of evidencebased perioperative care and stimulated further clinical research. This progressive shift illustrates how pragmatic initiatives, once validated and disseminated, may serve as bridges between expert consensus and guideline-level evidence^1^. 

 A growing number of guidance documents now use mixed strategies, combining systematic evidence synthesis with predefined consensus protocols (S3 classification according to AWMF). This approach seeks to preserve methodological rigor while allowing expert interpretation in areas with incomplete evidence. The Miami International Evidence-based Guidelines on Minimally Invasive Pancreas Resection exemplify this trend, integrating evidence-based methodology with Delphi-based agreement to formulate practical recommendations for complex surgical procedures^3^. 

 No single format is universally superior. Evidence-based guidelines are most appropriate when robust data exist and when clinical questions can be addressed through reproducible methodology. Structured consensus becomes valuable when evidence is limited or inconsistent, particularly in surgical fields where randomized trials are not feasible. Position papers help delineate concepts and institutional strategies. Protocols provide structured pathways and, when prospectively validated, may evolve into more mature evidence-based frameworks. Digestive surgery continues to advance rapidly, and the tools used to guide its practice must keep pace. From expert opinion to international guidelines, each format holds its place and must be constructed with methodological clarity and explicit acknowledgment of its limitations. When critically appraised and responsibly applied, group-based publications can strengthen clinical judgment, encourage investigation, and ultimately improve patient outcomes. 

 Looking forward, guidance documents in digestive surgery are expected to incorporate resources that enhance reproducibility, efficiency, and relevance. Artificial intelligence will likely assist in literature screening and preliminary risk-of-bias assessment, reducing the time and effort required to develop evidence-based guidelines and facilitating timely updates. Realworld data from hospital networks and national registries may complement trial data, where randomized evidence remains scarce. Living systematic reviews — with continuously updated evidence rather than static publication cycles — offer a model better suited to rapidly evolving topics and may eventually integrate directly into clinical guidance platforms. The use of transparent repositories, with open access to voting data and documented disagreement, may increase trust in consensusbased recommendations. Meanwhile, established appraisal frameworks are likely to gain traction, promoting higher standards of reporting and ensuring that guidance documents can be appropriately evaluated. As these tools mature, publication bias may be reduced, reproducibility enhanced, and the adoption of best practices in surgical care achieved more promptly. 

## Data Availability

The datasets generated and/or analyzed during the current study are available from the corresponding author upon reasonable request.
